# The role of dendritic cells and their interactions in the pathogenesis of antibody-associated autoimmune encephalitis

**DOI:** 10.1186/s12974-021-02310-z

**Published:** 2021-11-08

**Authors:** Fatme Seval Ismail, Sven G. Meuth, Nico Melzer

**Affiliations:** 1 Department of Neurology, University Hospital Knappschaftskrankenhaus Bochum, Ruhr University Bochum, In der Schornau 23-25, 44892 Bochum, Germany; 2 Department of Neurology, Heinrich-Heine University of Düsseldorf, Düsseldorf, Germany

**Keywords:** Dendritic cells, B cells, T cells, Antibodies, Autoimmune encephalitis

## Abstract

Autoimmune encephalitis (AE) is an inflammatory brain disease which is frequently associated with antibodies (Abs) against cell-surface, synaptic or intracellular neuronal proteins. There is increasing evidence that dendritic cells (DCs) are implicated as key modulators in keeping the balance between immune response and tolerance in the CNS. Migratory features of DCs to and from the brain are linked to initiating and maintaining of neuroinflammation. Genetic polymorphisms together with other triggers such as systemic or cerebral viral infection, or systemic malignancies could contribute to the dysbalance of “regulatory” and “encephalitogenic” DCs with subsequent dysregulated T and B cell reactions in AE. Novel in vivo models with implantation of mature DCs containing neuronal antigens could help to study the pathogenesis and perhaps to understand the origin of AE. Investigations of DCs in human blood, lymphoid tissues, CSF, and brain parenchyma of patients with AE are necessary to deepen our knowledge about the complex interactions between DCs, T and B cells during neuroinflammation in AE. This can support developing new therapy strategies.

## Background

Autoimmune encephalitis (AE) is an inflammatory brain disease which is frequently associated with antibodies (Abs) against neuronal cell-surface, synaptic or intracellular neuronal proteins [1–3]. Patients usually suffer from seizures, memory and cognitive deficits, behavioral changes, psychiatric symptoms, abnormal movements, dysautonomia, and a decreased level of consciousness [1, 2, 4]. Persons of all ages can be affected. Autoimmune disorders are the third most common cause of encephalitis [2, 5]. The anti-*N*-methyl-D-aspartate receptor (NMDAR) encephalitis is the most common form of AE and the anti-leucine-rich glioma-inactivated 1 (LGI1) AE the second most common form [6–8]. Further Abs associated with AE are directed against a variety of other surface membrane and synaptic membrane neuronal proteins such as α-amino-3-hydroxy-5-methyl-4-isoxazolepropionic acid receptor (AMPAR), γ-aminobutyric acid (GABA) type A or B receptor (GABA_A/B_R), contactin-associated protein-like 2 (CASPR2), dipeptidyl-peptidase-like protein 6 (DPPX), metabotropic glutamate receptor 5 (mGluR5), dopamine 2 receptor (D2R), neurexin-3α, or intracellular neuronal proteins such as Hu, Ma2, or glutamic acid decarboxylase 65 (GAD65) [1–4]. Systemic tumors and cerebral viral infections are potential trigger factors contributing to development of AE [1, 2]. Some of the tumors, e.g., small-cell lung cancer can express neuronal receptors [9] and others, e.g., ovarian teratoma can contain mature or immature nerve tissue [10, 11]. In case of the anti-NMDAR encephalitis, the neurons in the tumor express NMDAR similar to the neurons in the brain leading to an autoimmune response. In ovarian teratoma from patients with anti-NMDAR encephalitis, NMDAR-expressing neurons and inflammatory infiltrates of T and B lymphocytes were found, whereas in the tumors from patients without anti-NMDAR encephalitis only few or no B lymphocytes and plasma cells were detected [12]. In addition to this concept of classical onco-neuronal immunity where tumors and neurons express the same antigen, the concept of onco-neuronal cross-reactivity was recently shown likely to be relevant for the immunopathogenesis of anti-GABA_A_R encephalitis. Antibodies against the neuronal GABA_A_R protein were shown to recognize the onco-protein LIM-domain-only protein 5 (LMO5), which is related to cell-cycle regulation and tumor growth in a variety of hematological and solid tumors [13]. This suggests that expression of LMO5 in a tumor may initiate the formation of LMO5-directed antibodies that cross-react with the GABA_A_R and cause AE.

In vitro studies with cultured neurons showed that Abs can affect neuronal function through functional blocking of the target antigen (e.g., GABA_B_R), receptor cross-linking and internalization (e.g., NMDAR Abs), and disruption of protein–protein interactions (e.g., LGI1 Abs), potentially altering the function of the voltage-gated potassium channels and decreasing the levels of AMPAR [1, 2]. In a mouse model, the passive cerebroventricular transfer of Abs from the cerebrospinal fluid (CSF) of affected patients resulted in NMDAR internalization and impairment of long-term synaptic plasticity with gradual resolving of the alterations after cessation of the antibody infusion [14, 15]. In addition to these direct effects on neuronal function, depending on the Ig-subtype, antibodies may induce antibody-dependent cellular and complement-dependent cytotoxicity thereby destroying the target cells [16]. Moreover, initial evidence points towards a role of cytotoxic T cells in AE with Abs against surface membrane and synaptic membrane neuronal antigens [16, 17].

Abs against intracellular (so-called onco-neuronal) antigens in classical paraneoplastic AE are not directly pathogenic, and cytotoxic T cell mechanisms are thought to contribute to the pathogenesis [16, 18, 19]. It was suggested that antigens from apoptotic tumor cells are phagocytized and processed by dendritic cells (DC) (professional antigen-presenting cells (APCs)) that migrate to regional lymph nodes and present the antigens to CD4 + T cells, that license the DCs to cross-present the antigens to cytotoxic CD8 + T cells that become activated and contribute to an anti-tumor immune response [18, 19]. But this T cell-mediated autoimmune response is also directed against the same (onco-neuronal immunity) or a structurally related protein (onco-neuronal cross-reactivity) expressed in the brain with extensive infiltration of cytotoxic T cells and neuronal degeneration, e.g., via perforin/granzyme- or Fas-FasL-related mechanisms [18, 19]. In the regional lymph nodes, the antigen is also presented to naive B cells that with the help of CD4 + T cells become activated and differentiate into memory B cells on the one hand and antibody-producing plasma cells on the other hand that are capable of migrating into the CNS. In a model of NMDAR encephalitis, activated memory B cells have been shown to infiltrate the brain through crossing the choroidal plexus and undergo restimulation, antigen-driven affinity maturation, and differentiation into plasma cells [1, 2, 20]. It was suggested that a smaller amount of the Abs would cross the leaky or disrupted blood–brain barrier (BBB) [1, 2, 20]. In mGluR5-associated AE with Hodgkin lymphoma, the target antigen is not expressed by the tumor, and the mechanisms explaining the predominant association with Hodgkin lymphoma are unclear, but may be due to a paraproteinemic effect, i.e., malignant B lymphocytes secrete the antibody [1, 21]. On the other hand, onco-neuronal antigens are present in the tumor of patients with Ab-positive paraneoplastic AE, but also in many tumor patients without AE. Vice versa, in most patients with AE, no immunological triggers can be found and a genetic predisposition with HLA class II genes especially in anti-LGI1 encephalitis was reported [22, 23].

In summary, Ab-associated AE can occur independent of existing trigger factors. Further, the majority of patients with systemic tumor do not develop an autoimmune neurological disease [19]. The B and T lymphocytes are the mediators of autoimmunity, but their activation and function are under the control of DCs [24] which are present in lymphoid and non-lymphoid tissue as well as in the bloodstream. In other autoimmune CNS disorders such as multiple sclerosis (MS), DCs are involved in the regulation of autoimmune responses directed against myelin antigens through migration of immature DCs into the CNS where they are able to mature as well as present autoantigens to infiltrating T cells [25, 26]. Severity of experimental autoimmune encephalomyelitis (EAE), one of the experimental animal models of MS, as well as the number of MS plaques correlated with the functional state and compartmental distribution of DCs [27].

## Hypothesis: dendritic cells are implicated as key modulators in the pathogenesis of antibody-associated autoimmune encephalitis

Based on the above-mentioned findings and proposed models, DCs and their interactions may play a crucial role in loss of self-tolerance and initiation of CNS autoimmune inflammation in Ab-associated AE. Dendritic cells are ontogenetically and phenotypically a heterogeneous group of professional antigen-presenting cells which are potent inducers of immune responses by interaction with T cells [24]. These in turn can interact with other cells, such as B cells for antibody production, to complete the immune response. Functionally mature and immature DCs can be distinguished. Only mature DCs are able to activate T cells, but both immature and mature DCs can suppress T cell responses [27]. Two classes of DCs are distinguished by their localization, expression of surface proteins and functionality: plasmacytoid DCs (pDC), producing type I interferon and predominantly modulating immune response to viral infection by expressing, e.g., toll-like receptors (TLRs), and conventional DCs (cDC) as highly potent APCs, in particular for activating naive T cells [28]. The pCDs are present mainly in the blood and lymphoid tissues and under healthy conditions express low levels of major histocompatibility complex class II (MHC-II) and co-stimulatory molecules. In contrast, cDCs express high levels of MHC-II molecules [29]. Moreover, CNS-resident DC subsets are found in healthy brain tissue, meninges, choroid plexus and spinal cord, optimally localized to interact with infiltrating T cells since the choroid plexus, meninges and CNS parenchymal blood vessels are important entry portals for leukocytes during neuroinflammation [27, 30–32]. The origin of DCs within the CNS is widely debated and multiple mechanisms have been proposed such as recruitment from peripheral bone marrow-derived precursors, differentiation from resident microglia or from immature DCs in meninges or choroid plexus [30, 32]. It has been suggested that CNS-resident DCs may contribute to immune surveillance as well as autoimmune reaction with disease initiation [30, 32]. Unlike CNS-resident DCs, peripheral monocyte-derived DCs are not present under physiological conditions in the brain, but can differentiate from infiltrating monocytes and accumulate in the CNS during neuroinflammation [30]. Both DC subsets expanded in the meninges and CNS parenchyma during disease progression in EAE [30].

**Fig. 1:**
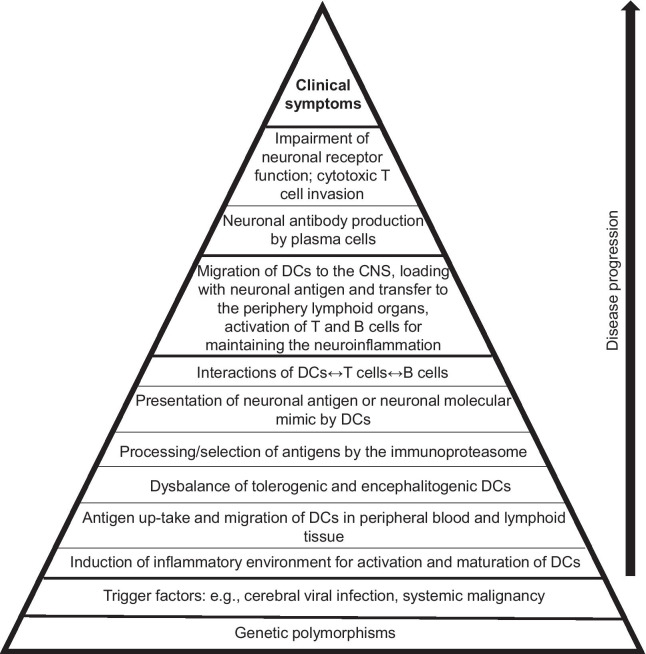
Proposed mechanisms involved in the pathogenesis of autoimmune encephalitis associated with neuronal antibodies. Genetic polymorphisms form the basis and the formation of antigen-specific cytotoxic T cells and antibodies together with local innate immune mechanisms lead to the formation of clinical symptoms as the “tip of the iceberg”. Dendritic cells (DCs) probably are key activators of the main players T and B cells

Further, DCs can produce different cytokines involved in the generation of effector and regulatory T cell responses against self- and non-self-antigens. Thus, the activation and maturation status of the DCs influences the induction of self-tolerance or immunity. We assume that a dysbalance of “regulatory” and “pro-inflammatory/encephalitogenic” DCs with dysfunction of their interactions with the T and indirectly also B cells may lead to disease pathogenesis in AE. Genetic polymorphisms together with other triggers such as cerebral viral infection, or systemic malignancies could contribute to the dysbalance and dysfunction of DCs and their interactions with T and B cells. Figure 1 summarizes the proposed mechanisms involved in the pathogenesis of Ab-associated AE.

Under physiological conditions, regulatory DCs downregulate immune responses by expressing a distinct combination of co-stimulatory and co-inhibitory surface molecules that promote immune tolerance and regulatory T cell (Treg) development. Antigen presentation in the absence of co-stimulatory signals or cytokines can promote the generation of anergic or immunosuppressive T cells. Therefore, DC presence in the CNS might limit neuroinflammation during CNS autoimmunity [33]. On the other hand, exacerbation of EAE associated with accumulation of CD4 + and CD8 + T cells in the CNS and disruption of the balance between encephalitogenic and regulatory T cells was observed after intracerebral injections of bone marrow-derived DCs loaded with myelin oligodendrocyte glycoprotein (MOG) peptide [34].

The cytokines interleukin 10 (IL-10) and interleukin 27 (IL-27) may play a role in promoting DC tolerogenic function [29]. IL-10-treated DCs induced anergy in alloantigen- and peptide-activated T cells [29, 35]. Previous in vitro studies demonstrated that a subset of conventional CD11c + DCs expressing perforin enforces peripheral tolerance by deleting T cells. Another subset of cDCs with tolerogenic properties is the population of CD103 + DCs in the intestinal mucosa [29]. The DC–T cell communication is complex, bidirectional, and mediated by soluble or cell surface molecules/receptors. In addition to the MHC peptide (I or II) and TCR-CD3-CD4/8 complex, B7-1 (CD80) and B7-2 (CD86) molecules on DCs bind to either CD28 or cytotoxic T-lymphocyte-associated Protein 4 (CTLA-4) to induce effector and suppressor functions in T cells, respectively. The classical B7/CD28 pathway leads to co-stimulation, but also inhibitory B7 family members including B7-H1 and B7-DC (programmed death-ligand 1 and 2 (PDL1 and PDL2), respectively) are expressed on mature DC and can contribute to inducing T cell tolerance by binding to the PD-1 receptor [27, 36]. Predominantly co-stimulatory interactions of DCs and T cells are probably also involved in the pathomechanism of AE.

There is evidence that increased numbers of activated DCs can be found in peripheral blood and cerebrospinal fluid (CSF) of patients with neuroinflammation including MS, suggesting their active participation in the immunopathogenesis [37, 38]. In correlation with these results, active recruitment into and accumulation of DCs in CNS white matter lesions and leptomeninges of patients with MS were demonstrated [26, 27, 39]. Interestingly, short-term treatment with high-dose intravenous methylprednisolone during MS relapse resulted in an increased number of Treg cells and reduced number of DCs in the blood correlating with clinical improvement [40]. Further findings showed impaired maturation and altered regulatory function of DCs in MS patients, suggesting other mechanisms of immune dysregulation [40, 41]. Treatment with glatiramer acetate partially restored phenotype and function of DCs [41]. In addition, DCs containing brain-derived antigens were discovered in the lymph nodes of MS patients supporting the hypothesis that during neuroinflammation, DCs can migrate to the periphery with CNS autoantigens and activate naive T cells [42], and these in turn can interact with B cells promoting antibody production. This may play a role in the maintenance of neuroinflammation during CNS autoimmunity also in Ab-associated AE.

Moreover, an important role in induction of self-tolerance may play the immunoproteasome in DCs, a multi-catalytic protease complex processing a number of antigenic peptides. The DCs expressing the immunoproteasome do not present peptides expressed in tumor as well as in healthy cells, allowing the escape of self-antigens from autoimmune damage [43]. The selection of some antigens by the immunoproteasome in DCs could provide new insights into the pathomechanisms during CNS autoimmunity in Ab-associated AE.

In conclusion, we hypothesize that DCs play a crucial, dominant role in the balance between immune response and tolerance during the development and progression of AE. Figure 1 sets out the causal chain thought to culminate in AE. Even if some links in the chain are optional, as in the case of infections or malignancy, the step with the interactions of DCs, T and B cells is indispensable. In our view, the DCs and their interactions are key, master controlling factors in all forms of AE.

## Implications of the hypothesis

With increasing evidence, DCs are implicated as key modulators in CNS immunity. Thus, explaining the migratory routes of DCs to and from the brain and the mechanisms of modulation of CNS immune responses by DCs could contribute to understand initiating and maintaining the CNS autoimmunity and developing new therapy strategies. In addition to previously known animal models [44], novel in vivo models of AE with implantation of mature DCs containing neuronal antigens could help to study the pathogenesis and perhaps to understand the origin of the disease. Thus, next steps include modifications of DCs by neuronal antigen-encoding mRNA or viral vectors that are able to induce co-stimulatory signals and antigen presentation leading to disease initiation after systemic administration in a novel mouse model. Investigations of the activation status of T cells and formation of Ab-producing B cells as well as histological evidence of CNS inflammation can confirm that pathogenesis and progression of AE are regulated by DCs. In addition, investigations of DCs in human blood, lymphoid tissues, and CSF as well as brain parenchyma in patients with AE according to MS studies are necessary to deepen our knowledge about the disease [37]. The immunological profile of human DCs obtained from therapy-naive patients needs to be specified using different methods such as flow cytometry, single-cell sequencing and analysis, immunohistochemical studies. This can be also useful to study ex vivo the complex interactions between DCs, T and B cells during neuroinflammation.

Moreover, a recent study showed that systemic delivery of nucleoside-modified, auto-antigen-encoding mRNA into lymphoid tissue-resident CD11c + APCs induced antigen-specific tolerance as a therapeutic approach in mouse models of MS [45]. Non-inflammatory mRNA encoding neuronal antigens such as NMDAR, LGI1 or others could offer a new treatment option also for the group of Ab-associated CNS autoimmunity [46]. Dendritic cells as APCs exposed to auto-antigen-encoding, non-inflammatory mRNA could induce antigen-specific T cell tolerance to avoid B cell activation and differentiation into Ab-producing plasma cells, supporting our hypothesis that DCs are key modulators in the immunopathogenesis of Ab-associated AE, but also maybe key players in future treatment perspectives.

## Data Availability

Not applicable.

## Abbreviations

AMPAR: α-Amino-3-hydroxy-5-methyl-4-isoxazolepropionic acid receptor
APC: Antigen-presenting cell
AE: Autoimmune encephalitis
Ab: Antibody
BBB: Blood–brain barrier
CSF: Cerebrospinal fluid
CNS: Central nervous system
CASPR2: Contactin-associated protein-like 2
cDC: Conventional dendritic cell
CTLA-4: Cytotoxic T-lymphocyte-associated Protein 4
DC: Dendritic cells
DPPX: Dipeptidyl-peptidase-like protein 6
D2R: Dopamine 2 receptor
EAE: Experimental autoimmune encephalomyelitis
GABA: γ-Aminobutyric acid
GAD65: Glutamic acid decarboxylase 65
Ig: Immunoglobulin
IL: Interleukin
LGI1: Leucine-rich glioma-inactivated 1
MHC: Major histocompatibility complex
mGluR5: Metabotropic glutamate receptor 5
MOG: Myelin oligodendrocyte glycoprotein
mRNA: Messenger-RNA
MS: Multiple sclerosis
NMDAR: *N*-Methyl-d-aspartate receptor
pDC: Plasmacytoid dendritic cell
PDL: Programmed death-ligand
TLR: Toll-like receptor
Treg: Regulatory T cell
